# The expression of glycolysis-related proteins in urine significantly increases after running

**DOI:** 10.3389/fphys.2024.1481741

**Published:** 2024-12-09

**Authors:** Tian Zhao, Tianci Liu, Tao Li, Shengcun Chen, Lupeng Wang, Man Zhang

**Affiliations:** ^1^ College of Information Engineering, Hangzhou Dianzi University, Hangzhou, China; ^2^ Beijing Key Laboratory of Urinary Cellular Molecular Diagnostics, Beijing, China; ^3^ Clinical Laboratory Medicine, Beijing Shijitan Hospital, Capital Medical University, Beijing, China; ^4^ Institute of Regenerative Medicine and Laboratory Technology Innovation, Qingdao University, Qingdao, China

**Keywords:** urine, glycolysis-related proteins, running, training, biomarkers

## Abstract

**Objective:**

Glucose metabolism is the main way in which cells obtain energy during exercise and plays an important role in exercise. The purpose of this study was to explore the changes in the expression of glucose metabolism-related proteins in urine after running, and finally applied to the monitoring of running training.

**Methods:**

Urine samples were collected before and after running, and urine proteomics information was collected to explore the expression of proteins in the urine using LC-MS/MS in DDA mode and DIA mode. Receiver operating characteristic (ROC) curve was drawn to evaluate the value of target proteins in monitoring running training.

**Results:**

A total of 140 proteins were identified using LC-MS/MS in DDA mode, of which 49 urine proteins showed increased expression after running. KEGG analysis revealed that glucose metabolism-related proteins are mainly concentrated in glycolysis. There were six glycolysis-related proteins, among which urine proteins PKM, TPI1, ENO1 and LDHB were significantly increased after running (*P* < 0.05). This changes in urine proteins PKM, TPI1, ENO1 and LDHB were further verified by the results of LC-MS/MS in DIA mode. The concentrations of the urine proteins TPI1, ENO1 and LDHB showed a significant linear relationship with PKM. ROC curve analysis showed that PKM, TPI1, ENO1 and LDHB proteins in urine had good monitoring values for running training.

**Conclusion:**

The expression of glycolysis-related proteins PKM, TPI1, ENO1 and LDHB in urine was significantly increased after running, which may be applied to the monitoring of running training.

## Introduction

With the development of sports science, sports training monitoring is receiving increasing attention and recognition, and has become a research hotspot. At present, various physiological and biochemical indicators are used to monitor exercise training, such as serum creatine kinase, blood lactate, urine albumin, etc. (Asghar et al., 2017; Benedict et al., 2011). The commonly used samples are blood and urine. Compared with blood, urine is convenient for sampling, harmless, easy to be measured repeatedly during exercise (Aitekenov et al., 2021; Capolongo et al., 2018), and more sensitive and earlier to reflect the degree of exercise training. Urine has become the sample of choice in sports training monitoring.

In recent years, with the development of urine proteomics, the analysis and application of urine metabolites for sports training monitoring has also been put on the agenda (Contrepois et al., 2020; Heaney et al., 2019; Quintas et al., 2020). Proteomics research has the characteristics of high throughput, high efficiency, accuracy and sensitivity (Khan et al., 2020), which can study the relationship between exercise and urine protein components from an overall perspective. Training effectiveness and fatigue injury can be effectively assessed and monitored by studying the components of urine proteins in different sports and identifying the characteristic proteins. Many scholars have tried to apply proteomics technology to exercise research, including acute exercise, long-term exercise and marathon. This approach has proven to be a very effective method of research (Enea et al., 2009; Zhu et al., 2021). For example, Samudrala et al. applied mass spectrometry technology to detect urine protein and proved that urine protein components can more comprehensively reflect the basic status of body metabolism after athletes exercise (Samudrala et al., 2015).

Among various metabolic processes, glucose metabolism can provide the carbon skeleton and energy for cellular life activities, and its homeostasis is fundamental to the physiological function of each tissue of the body (Mulukutla et al., 2016). Glucose is the main energy supply substance in the body, and the energy consumed during exercise is mainly derived from glucose. Its metabolic disorders are closely related to the occurrence of metabolic diseases such as obesity and type Ⅱ diabetes (Bouché et al., 2013; Norton et al., 2022). Glucose metabolism is the process by which the body carries out orderly chemical reactions for the absorption and utilization of glucose. The main pathways are anaerobic glycolysis, aerobic oxidation, and the pentose phosphate pathway. Good glucose metabolism improves the body’s energy supply, which in turn improve exercise performance. Until now, the expression and significance of urine glucose metabolism-related proteins during exercise has not been particularly well understood and is therefore of great importance for the monitoring of glucose metabolism during exercise.

In this study, a mass spectrum database of urine proteins of subjects before and after running was established using proteomic methods. The expression changes of glucose metabolism-related proteins in urine were analyzed and their monitoring effect on running training was evaluated, thus providing a suitable urine biomarker for running training monitoring.

## Materials and methods

### Subjects

Twenty males from Beijing Shijitan Hospital affiliated to Capital Medical University were enrolled as subjects. A 2000 m endurance run at a constant speed of 9 km/h was used during the running. Informed consent was obtained from all subjects prior to inclusion in this study. All procedures were performed in accordance with the ethical standards of the Declaration of Helsinki and approved by the Ethics Committee of Beijing Shijitan Hospital. In order to ensure the consistency of sampling before and after the experiment, the sampling requirements (diet, etc.), sampling time points, sampling methods, and sampling procedures were introduced to the subjects in advance.

### Sample collection and processing

30 mL of urine was collected from the subjects 10 min before and 10 min after running. The urine sample was divided into two parts, one of which was packaged and stored at −80°C until used for mass spectrometry detection. Another part of the sample was used for urine routine testing. Urine routine testing was performed using the MUS9600 Urine Analyzer (Dirui, Changchun, China), which was completed within 2 h after sampling.

### Urine sample preparation for MS analysis

1 mL was removed from the urine sample and the pellet were collected by centrifugation at 176,000 *g* for 1 h. The pellet was resuspended with 40 μL of resuspension buffer (50 mM Tris, 250 mM sucrose, pH 8.5) and 50 mM dithiotheitol (DTT), after which it was heated at 65°C for 30 min. Then 160 μL of washing buffer (100 mM NaCl, pH 7.4) was added, and a second ultracentrifugation was performed at 176,000 g for 30 min. The pellet was resuspended in 30 μL NH_4_HCO_3_ and heated at 95°C for 3 min. It was cooled to room temperature and digested with trypsin for 12 h. It was then dried in vacuum and re-dissolved in 0.1% formic acid. And resolved on an UltiMate 3000 RSLCnano System (Thermo Fisher Scientific) at a flow rate of 800 nL/min over a 30-min linear gradient (5%–35% acetonitrile in 0.1% formic acid).

### LC-MS/MS in data-dependent acquisition mode

The samples were loaded onto the trap column (100 μm * 2 cm, homemade; particle size, 3 μm; pore size, 120 A; SunChrom, United States). With a gradient of 5%–35% mobile phase B (20%H_2_0 and 80%ACN, 0.08%FA) at a flow rate of 800 nL/min for 30 min, peptide samples were separated by a homemade silica microcolumn (150 μm * 10 cm, particle size, 1.9 μm; pore size, 120 A. SunChrom, United States). LC-MS/MS was performed on a Q Exactive HF-x mass spectrometer (Thermo Fisher Scientific). The instrument was operated in data-dependent acquisition mode (DDA). Full scans were performed from m/z 300 to 1,400.

The MS data was processed on the Firmiana platform. Proteins were identified from the NCBI human RefSeq protein database. 1% FDR was allowed on both the peptide and protein levels estimated by searching a decoy database. Only identifications with >l unique and strict peptides and >2 strict peptides (ion score >20) or >3 strict peptides, which was comparable to 1% FDR at the protein level, were used for subsequent analysis. For protein quantification, intensity based absolute quantification (iBAQ) algorithm was used. To normalize differences in sample size, iBAQ values were converted to iFOT (fraction of total) calculated by dividing the iBAQ value of each protein by the total iBAQ of the sample followed and multiplying 10^5^ for easy visualization (Hadjadj et al., 2020). Finally, based on the online resource database Kyoto Encyclopedia of Genes and Genomes (KEGG: https://www.genome.jp), the protein enrichment degree in the KEGG Pathway was evaluated using R language and hypergeometric test.

### LC-MS/MS in data-independent acquisition mode

Experiments were performed on a QExactive HF-X mass spectrometer (Thermo Fisher), and data-independent acquisition (DIA) mode was used for mass spectrometry with a full scanning range of m/z 350–1,500 and a primary mass resolution of 120,000. The “Top Speed” mode was adopted for secondary mass spectrometry detection, with the resolution set at 30,000, AGC at 100%, maximum injection time at 54 ms. Finally, the raw data of mass spectrometry detection was generated. Mass spectrometry results were queried in the NCBI human RefSeq protein database. At the protein level, each protein contained at least one unique peptide using a 1% FDR as a filter. Proteins with a fold change >1.5 and p value <0.05 were considered significantly different.

### Statistics

Statistical analysis was performed using GraphPad Prism 8.0 (GraphPad, La Jolla, CA, United States) software, and differences between groups were compared using the Student’s t-test. Correlation analysis was determined using the Pearson correlation. Receiver operating characteristic (ROC) curve analysis was used to evaluate the diagnostic performance of the two groups of proteins. *P* < 0.05 was considered significant and was considered statistically significant.

## Results

### Clinical characteristics

The basic information of the selected 20 subjects was shown in Table 1, the age was (25.80 ± 1.91) years old, the height was (1.73 ± 0.07) m, the weight was (71.48 ± 10.59) kg, and the BMI was (23.86 ± 3.00) kg/m^2^. The details of the 20 subjects can be found in Supplementary Table S1.

**TABLE 1:** Basic information of subjects.

Number	Gender	Age (years)	Height (m)	Weight (kg)	BMI (kg/m^2^)
20	Male	25.80 ± 1.91	1.73 ± 0.07	71.48 ± 10.59	23.86 ± 3.00

### Changes in subject indicators

The changes in blood pressure, heart rate, blood glucose levels and urine routine before and after running were shown in Table 2. According to the table, the systolic blood pressure and heart rate of the subjects increased significantly and blood glucose decreased significantly after running. There were no significant changes in urine pH, urine specific gravity (SG). There were no positive cases of urine protein and urine glucose before or after running.

**TABLE 2:** Subject measurement indicators.

Characteristics	Before running	After running	P
systolic blood pressure (mmHg)	122.15 ± 10.86	135.40 ± 12.60	***
diastolic blood pressure (mmHg)	80.25 ± 9.35	75.90 ± 11.66	ns
heart rate (times/min)	74.00 ± 10.55	122.95 ± 14.02	***
blood glucose (mmol/L)	5.51 ± 0.33	5.17 ± 0.45	**
pH	6.35 ± 0.56	6.08 ± 0.47	ns
urine protein (%)	0	0	NA
urine glucose (%)	0	0	NA
SG	1.02 ± 0.01	1.02 ± 0.01	ns

SG: Specific Gravity. ns, no significance; *, *P* < 0.05; **, *P* < 0.01; ***, *P* < 0.001.

### Proteomic analysis of urine proteins

**FIGURE 1:**
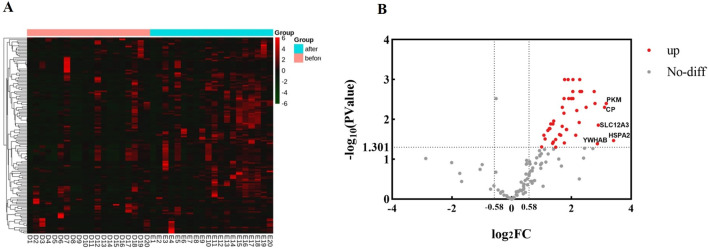
Proteomic analysis of urine proteins. The analysis was carried out according to the mass spectrometry data in DDA mode. **(A)** Clustering heatmap analysis of proteins in urine before and after running. before, group before running; after, group after running. **(B)** Volcano diagram analysis of urine proteins before and after running. The abscissa is denoted by log_2_ (FC), and the ordinate is denoted by −log_10_ (P Value).

140 proteins were identified in urine by LC-MS/MS in data-dependent acquisition mode (DDA), and the clustering heatmap of these proteins was shown in Figure 1A. Fold change >1.5 and P value <0.05 were considered as significant differences. The volcano diagram was shown in Figure 1B, where 49 proteins were elevated in the urine after running compared to the urine before running.

### KEGG analysis of urine proteins

**FIGURE 2:**
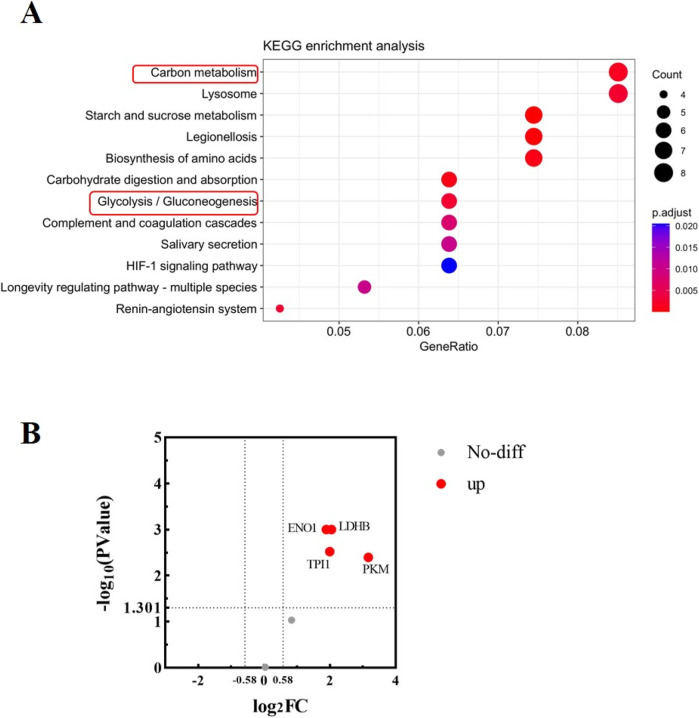
Bioinformatics analysis of urine proteins. **(A)** KEGG enrichment analysis of urine proteins. The analysis was performed based on 140 proteins obtained by mass spectrometry. The vertical axis represents significantly enriched KEGG pathways, the horizontal axis represents the proportion of proteins, the size of the dot represents the number of genes, and the color represents the magnitude of the p value. **(B)** Volcano diagram analysis of glycolysis-related proteins in urine before and after running. The abscissa is denoted by log_2_ (FC), and the ordinate is denoted by −log_10_ (P Value).

To further explore the function of 140 proteins in urine, a KEGG analysis was performed. As shown in Figure 2A, most of the proteins were aggregated during the processes of Carbon metabolism, Starch and sucrose metabolism, indicating vigorous metabolism during running. Glycolysis was the main aggregation pathway for glucose metabolism, with a total of six proteins aggregating in the glycolysis pathway, including TPI1, PKM, LDHB, GAPDH, ENO1 and ALDOB. Fold change >1.5 and P value <0.05 were considered as significant differences, and the volcano diagram was shown in Figure 2B. Compared with those before running, the expressions of glycolysis-related proteins GAPDH and ALDOB in urine after running did not change significantly, while expression of PKM, TPI1, ENO1 and LDHB proteins increased.

### Changes in the expression of glycolysis-related proteins in the urine of subjects after running

**FIGURE 3:**
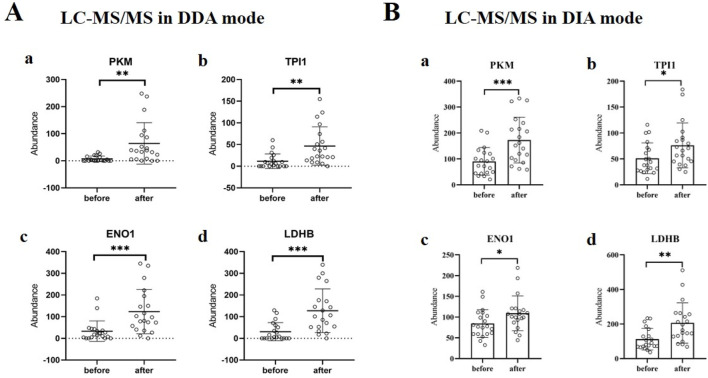
Changes in the expression of glycolysis-related proteins in urine of subjects after running. **(A)** The expressions of PKM (a), TPI1 (b), ENO1 (c) and LDHB (d) in urine before and after running. The analysis was carried out according to the mass spectrometry data in DDA mode. **, *P* < 0.01; ***, *P* < 0.001. **(B)** The expressions of PKM (a), TPI1 (b), ENO1 (c) and LDHB (d) in urine before and after running. The analysis was carried out according to the mass spectrometry data in DIA mode. *, *P* < 0.05; **, *P* < 0.01; ***, *P* < 0.001.

According to the mass spectrometry data in DDA mode, it can be found that the expression levels of PKM, TPI1, ENO1 and LDHB proteins in urine after running were significantly higher than those before running (*P* < 0.05), and the difference was statistically significant (Figure 3A). Meanwhile, the mass spectrometry data in DIA mode also demonstrated the increased expression of PKM, TPI1, ENO1, and LDHB in urine after running (Figure 3B).

### Correlation analysis of urine glycolysis-related proteins

**FIGURE 4:**
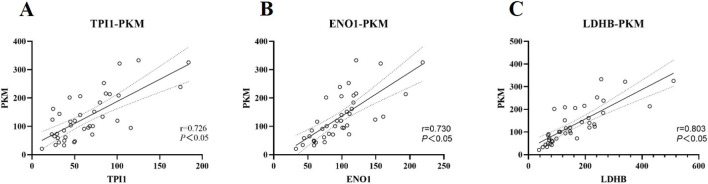
Correlation analysis of urine TPI1, ENO1, LDHB and PKM expression. The analysis was carried out according to the mass spectrometry data in DIA mode. **(A)** Correlation analysis of urine TPI1 protein and PKM protein. r = 0.726, *P* < 0.05. **(B)** Correlation analysis of urine ENO1 protein and PKM protein. r = 0.730, *P* < 0.05. **(C)** Correlation analysis of urine LDHB protein and PKM protein. r = 0.803, *P* < 0.05.

The proteins PKM, TPI1, ENO1 and LDHB are important catalytic enzymes in the glycolytic pathway and there should be some correlation between them. Based on the mass spectrometry data in DIA mode, the correlation between urine proteins PKM, TPI1, ENO1 and LDHB was further explored, as shown in Figure 4. The concentrations of urine proteins TPI1, ENO1 and LDHB were linearly correlated with PKM (*P* < 0.05).

### Urine glycolysis-related proteins have auxiliary monitoring value for running training

**FIGURE 5:**
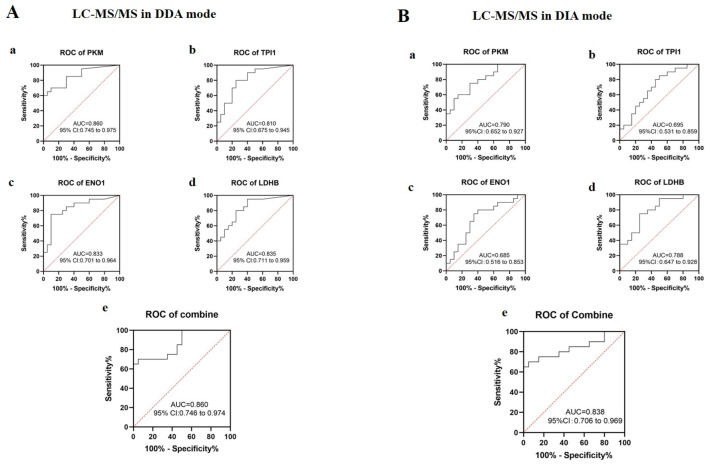
ROC curve analysis of urine glycolysis-related proteins in running training monitoring. **(A)** ROC curves were drawn based on the abundance values of the target proteins in urine from the mass spectrometry data in DDA mode. a: PKM; b: TPI1; c: ENO1; d: LDHB; e: four proteins in combination. AUC, area under the curve; CI, confidence interval. **(B)** ROC curves were drawn based on the abundance values of the target proteins in urine from the mass spectrometry data in DIA mode. a: PKM; b: TPI1; c: ENO1; d: LDHB; e: four proteins in combination. AUC, area under the curve; CI, confidence interval.

Based on the mass spectrometry data, ROC curves were established to analyze the auxiliary monitoring values of the urine proteins PKM, TPI1, ENO1, and LDHB for running, as shown in Figure 5. According to the mass spectrometry data in DDA mode (Figure 5A), the area under the curve (AUC) of urine protein PKM was 0.860. The AUC of urine protein TPI1 was 0.810. The AUC of urine protein ENO1 was 0.833. The AUC of urine protein LDHB was 0.835. When the four urine proteins were combined, the AUC was 0.860. According to the mass spectrometry data in DIA mode (Figure 5B), the AUC of urine protein PKM was 0.790. The AUC of urine protein TPI1 was 0.695. The AUC of urine protein ENO1 was 0.685. The AUC of urine protein LDHB was 0.788. When the four urine proteins were combined, the AUC was 0.838. These four glycolysis-related proteins are of good application value and can be used for the auxiliary monitoring of running training.

## Discussion

Glucose metabolism plays an important role in maintaining the energy homeostasis and growth and development of the human body. Exercise training monitoring is a hot topic in current exercise science research. Exploring changes in the expression of glucose metabolism-related proteins during exercise is of great significance for the selection of biomarkers for exercise training monitoring.

In this study, we investigated the changes of glucose metabolism-related proteins in urine before and after running. It was found that glucose metabolism was mainly concentrated in the glycolytic pathway during running, and expression of glycolysis-related proteins PKM, TPI1, ENO1 and LDHB increased significantly after running. The expression of urine PKM, TPI1, ENO1 and LDHB proteins increased after running, which may have applications as potential biomarkers for running training monitoring.

As an ultrafiltrate of plasma, urine is the end product of systemic organ metabolism (Zhou et al., 2019). Changes in urine protein expression should be consistent with systemic protein expression. Changes in blood glucose concentration are closely related to glucose metabolism. After the running, the subjects showed a significant decrease in blood glucose concentration, indicating that glucose metabolism was vigorous and providing sufficient energy for the exercise. Glycolysis is the process by which glucose is broken down to pyruvate, which produces ATP. As a continuous process, abnormalities in all stages of glycolysis can lead to disturbances in energy supply. Glycolysis-related proteins are important catalytic enzymes in the process of glycolysis, and they are closely related to each other. This association was further validated by the significant positive correlation between urinary concentrations of the proteins TPI1, ENO1, and LDHB and PKM.

During glycolysis, the TPI1 protein of these four proteins was the first to become functional.TPI1 (Triosephosphate isomerase) is involved in the glycolytic pathway by catalyzing the interconversion of glyceraldehyde triphosphate and glycerone diphosphate (Jin et al., 2022). It regulates the balance of three-carbon glucose metabolism in cells, and directly affects ATP production and cellular energy supply. ENO1 (Alpha-enolase), also known as 2-phospho-D-glycerate hydrolase, catalyzes the conversion of 2-phosphoglycerate to phosphoenolpyruvate during glycolysis. ENO1 is ubiquitously expressed in most human tissues and is overexpressed in multiple cancer types (Huang et al., 2022). PKM (Pyruvate kinase) is one of the major rate-limiting enzymes in glycolysis and is widely present in muscle and liver (Damasceno et al., 2020; Dayton et al., 2016). Pyruvate kinase encoded by PKM is responsible for catalyzing the last step of the glycolytic pathway, which is the transfer of a phosphoryl group from phosphoenolpyruvate to ADP, resulting in the production of pyruvate and ATP. Pyruvate is subsequently reduced to lactate by lactate dehydrogenase (LDH) in the cytosol or is directed by the respiratory chain to produce ATP in high yields (Nicholas et al., 2015). LDH, as a key enzyme in glycolysis, has two types: LDHA and LDHB. LDHB is widely found in the kidney, myocardium and skeletal muscle (Fan et al., 2021). In addition, studies have shown that LDHB is localized to mitochondria and is involved in a variety of key cellular processes, including ATP production, apoptosis, calcium homeostasis, and cell proliferation (Lovas and Wang, 2013; Mishra and Chan, 2014). In general, PKM, TPI1, ENO1 and LDHB are closely related to each other and play important roles in energy metabolism and ATP production during glycolysis.

In this study, we found that glucose metabolism is mainly concentrated in the glycolytic pathway, which may be related to the form of running, with the body’s glycolysis utilization being relatively high during the 2000 m medium and long distance runs. After running, the concentrations of urine PKM, TPI1, ENO1, and LDHB proteins increase, indicating that the body’s glucose metabolism is vigorous and the glycolysis energy supply is increased. Therefore, the monitoring of glycolysis-related proteins is of great importance.

This study is the first to report the expression of glycolysis-related proteins in urine before and after running. Urine sample collection is simple and non-invasive, which can meet the needs of running training monitoring biomarkers (Castaño et al., 2019). The expressions of urine glycolysis-related proteins PKM, TPI1, ENO1 and LDHB are increased after running, which are expected to become potential biomarkers for running training monitoring. Of course, current research on urine glycolysis-related proteins before and after running is still in the preliminary exploration stage, and needs to be evaluated and validated in more forms of exercise and in more subjects.

## Conclusion

In this study, we explored the changes in the expression of urine glycolysis-related proteins in normal subjects before and after running using mass spectrometry. Glucose metabolism is enhanced after running, and the concentrations of urine glycolysis-related proteins PKM, TPI1, ENO1 and LDHB are increased, which can be used as potential biomarkers for running training monitoring, providing a promising method for non-invasive running training monitoring.

## Data Availability

The data analyzed in this study is subject to the following licenses/restrictions: The datasets generated and analysed during the current study are not publicly available but are available from the corresponding author on reasonable request. Requests to access these datasets should be directed to zhangman@bjsjth.cn.
