# Supplementary material for: The expression of glycolysis-related proteins in urine significantly increases after running
Source: Front Physiol. 2024 Dec 9;15:1481741. doi: 10.3389/fphys.2024.1481741 (PMC11663847; doi:10.3389/fphys.2024.1481741)
Supplement: Supplementary file 1 [file Table1.docx]

**Table S1 Specific information of 20 subjects**

| Number | Gender | Age (years) | Height (m) | Weight (kg) | BMI（kg/m^2^） |
| --- | --- | --- | --- | --- | --- |
| 1 | male | 30 | 1.70 | 80.00 | 27.68 |
| 2 | male | 29 | 1.73 | 77.20 | 25.79 |
| 3 | male | 28 | 1.77 | 65.00 | 20.75 |
| 4 | male | 28 | 1.80 | 75.00 | 23.15 |
| 5 | male | 27 | 1.81 | 87.00 | 26.56 |
| 6 | male | 26 | 1.65 | 50.00 | 18.37 |
| 7 | male | 26 | 1.80 | 93.00 | 28.70 |
| 8 | male | 26 | 1.65 | 70.00 | 25.71 |
| 9 | male | 26 | 1.72 | 65.00 | 21.97 |
| 10 | male | 26 | 1.68 | 76.00 | 26.93 |
| 11 | male | 25 | 1.60 | 57.50 | 22.46 |
| 12 | male | 25 | 1.70 | 85.00 | 29.41 |
| 13 | male | 25 | 1.73 | 60.00 | 20.05 |
| 14 | male | 25 | 1.78 | 78.00 | 24.62 |
| 15 | male | 25 | 1.68 | 69.00 | 24.45 |
| 16 | male | 25 | 1.75 | 70.00 | 22.86 |
| 17 | male | 25 | 1.85 | 73.30 | 21.42 |
| 18 | male | 24 | 1.83 | 73.55 | 21.96 |
| 19 | male | 23 | 1.62 | 60.00 | 22.86 |
| 20 | male | 22 | 1.74 | 65.00 | 21.47 |
